# Q&A: How can advances in tissue clearing and optogenetics contribute to our understanding of normal and diseased biology?

**DOI:** 10.1186/s12915-017-0421-3

**Published:** 2017-09-25

**Authors:** Alon Greenbaum, Min J. Jang, Collin Challis, Viviana Gradinaru

**Affiliations:** Division of Biology and Biological Engineering, California Institute of Technology, Pasadena, CA 91125 USA

## Abstract

Mammalian organs comprise a variety of cells that interact with each other and have distinct biological roles. Access to evaluate and perturb intact biological systems at the cellular and molecular levels is essential to fully understand their functioning in normal and diseased conditions, yet technical limitations have constrained most research to small pieces of tissue. Tissue clearing and optogenetics can help overcome this hurdle: tissue clearing affords optical interrogation of whole organs at the molecular level, and optogenetics enables the scalable control and measurement of cellular activity with light. In this Q&A, we delineate recent advances and practical challenges associated with these two techniques when applied body-wide.

## How can tissue clearing and optogenetics benefit the study of diseases?

Mammalian organs are highly coordinated, complex networks of cells. Our understanding of organ physiology and pathology would benefit from study of the structures and even organisms as a whole; however, technical limitations have historically forced most preclinical research to focus on small pieces of organ tissue, rather than intact organs. In neuroscience, efforts have been made to understand the brain as a whole system while maintaining cell and/or circuit resolution, resulting in substantial advances in technologies such as tissue clearing (TC), optogenetics, and gene delivery via viral vectors. Recent developments in TC have greatly increased our ability to interrogate whole organs—and even whole organisms—by rendering the tissue optically transparent. Compared with traditional techniques, this has the advantage of leaving complex intercellular pathways intact and retaining dispersed, subtle features [1–3]. This allows us not only to study the 3-D structure of normal tissue in unprecedented detail, but also to investigate sparsely distributed pathological hallmarks in disease models; for example, amyloid plaques in the brain of Alzheimer’s disease mouse models [4]. While TC has been instrumental in mapping the streets, so to speak, optogenetic tools are used to monitor and control the traffic, or activity, that uses these pathways. These methods are therefore used synergistically: TC is conducted post-mortem whereas optogenetics is a real-time technique that, defined broadly, uses light to either manipulate or report [5] neuronal activity in vivo. In combination with genetic targeting strategies, optogenetics allows researchers to study cellular populations with high temporal precision. Recently developed tools allow us to control and record from highly specific populations of neurons in broadly distributed neural networks [6]. This approach has revealed various functional circuits spanning across brain regions [7, 8], providing potential therapeutic targets for neurological disorders. Beyond neuroscience, TC and optogenetic techniques have enabled a new research pipeline for the study of diseases, allowing us to access and assess the anatomy and functions of a broad range of biological systems, including the peripheral nervous system, more precisely than before.

## There are so many tissue clearing methods, which one to use?

By and large, there is no ‘one size fits all’ method for TC—it is application dependent. Each clearing method has its own strengths and weaknesses, and many tradeoffs need to be considered, such as: compatibility with immunohistochemistry (IHC) and fluorescence in situ hybridization (FISH), long-term preservation of endogenous fluorescence, morphology changes (such as shrinking or expansion of the tissue), and clearing time. For a comprehensive comparison of TC methods refer to [1, 2, 9]. Given the variability in clearing results, we recommend trying a few TC methods in parallel and selecting the one that satisfies the experimental endpoints.

**Fig. 1.**
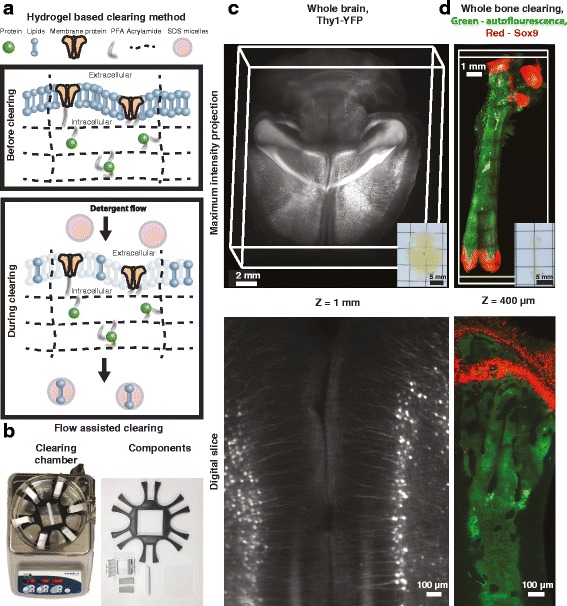
Hydrogel-based clearing methods and their application to whole-brain and intact-bone clearing. **a** The protein retention mechanism and the lipid removal process. Prior to lipid removal, the proteins are anchored to a hydrogel scaffold via PFA-mediated binding. During the clearing process, the detergent selectively removes light scattering lipids, while proteins remain locked in place by the hydrogel. Following lipid removal, the tissue is rendered transparent. **b** The clearing chamber and its components. With the use of continuous conductive flow, the clearing process is accelerated (~ 2–3× faster) and can clear a whole brain or an entire bone. From [17]. Reprinted with permission from AAAS. **c** Maximum intensity projection of a PACT-cleared whole mouse brain (*Thy1-YFP*) and a digital section (1 mm deep). **d** Maximum intensity projection of a mouse femur cleared using Bone CLARITY, and a digital section (0.4 mm deep). The *green* channel represents auto-fluorescence, while the *red* channel represents cells that express Sox9. The images in **c** and **d** were captured using a commercial and custom light-sheet microscope, respectively. From [17]. Reprinted with permission from AAAS

In our work, we typically utilize the passive clarity technique (PACT), which retains endogenous fluorescence [10, 11], is compatible with IHC and single molecule FISH (smFISH), and provides excellent clearing results. However, PACT can be a time-consuming method and the cleared tissue expands [10], a property that was also recruited by expansion microscopy [12], since this expansion can be controlled or amplified (ePACT) in beneficial ways as it also preserves endogenous fluorescence [11, 13]. PACT begins with paraformaldehyde (PFA) perfusion and post-fixation stages, followed by hydrogel embedding. When the hydrogel is polymerized, it acts as a scaffold and locks proteins, DNA, and RNA in place for subsequent detection (Fig. 1a). Because the light-scattering lipids are not anchored to the hydrogel, they can be removed by a relatively gentle detergent, sodium dodecyl sulfate (SDS), that leaves most epitopes and fluorescent proteins well preserved. The hydrogel composition is vital; it needs to be sufficiently dense to lock the proteins firmly, yet sparse enough to allow detergent to flow throughout the sample to wash away the opaque lipids. When using a continuous conductive flow (Fig. 1b), an entire adult mouse brain can be cleared and imaged using PACT (Fig. 1c).

## Can tissue clearing be applied to any organ?

Although TC techniques have been demonstrated mostly in the brain, all soft organs can be successfully cleared with various TC techniques, either by transcardial perfusion, in which the clearing reagents are delivered through the vasculature to the entire body [10, 11, 14], or by dissecting, extracting, and clearing a specific organ of interest. The extension of TC from the brain to the rest of the body has resulted in a myriad of applications, for example: profiling cancer metastasis in a whole organism [15] and studying axon regeneration in the peripheral nervous system (PNS) after spinal cord injury [16]. The PNS is essential for peripheral organ functions such as the control of hormone secretion in the pancreas and autonomous beating of the heart. Because peripheral nerve fibers densely innervate peripheral organs, TC techniques will greatly enhance the study of interactions between the PNS and organ functions, as well as related diseases.

Besides soft tissue, organs with unique structures and/or composition (such as bones) have recently been cleared with a modified TC technique (Bone CLARITY [17]; Fig. 1d; details discussed below). Amongst other benefits, this method can now enable clearing of the intact skull, allowing researchers to study not only the structure of the brain *per se,* but also the delicate interface between bone and brain, where the lymphatic vessels reside [18, 19].

## Can we apply tissue clearing techniques to study human organs?

Yes, this is possible, although factors regarding fixation, labeling, scalability, and adaptability must be considered. Typically, the first step in TC methods is to remove blood and fix the organs of the animal model via transcardial perfusion. During this process, formaldehyde is perfused through the vasculature of the post-mortem animal to prevent the tissue from decomposing. This makes it possible to achieve a uniform fixation and to reach difficult-to-access locations throughout the animal’s body. Since perfusion is not widely used in post-mortem humans and is challenging in excised tissue (from biopsies, for instance; the Langendorff is a niche option for explanted hearts from transplantation surgery [20]), immersion-based fixation is typically used. The immersion-based fixation method results in suboptimal preservation of proteins and epitopes, potentially causing the loss of biological signals, and difficulties in labeling of other significant biomolecules, especially RNA. These labeling issues are aggravated in tissues rich in enzymes/proteases such as the pancreas or gut. Also, existing human tissue is usually collected and stored flash frozen or as formalin-fixed, paraffin-embedded sections, which is not fully compatible with current TC and biomolecule labeling methods. Although a few successful attempts at human tissue clearing have been reported [11, 21], the formalin-based fixation of human tissue causes the TC and antibody labeling steps to be more time consuming.

Additional considerations concerning the distinct biological composition of human tissue compared with rodent tissue require further optimization of TC and staining procedures. For example, human brain samples require longer clearing time to become transparent than mouse brain samples with the same thickness [22]. Even though human tissue specimens acquired in clinical circumstances are often heterogeneous and fragile, embedding them in firming hydrogel and clearing is possible, even for challenging samples such as the pancreas [23]. However, some challenges to be aware of when working with human samples are: (1) residual blood autofluorescence, which makes imaging difficult; (2) suboptimal fixation (under or over crosslinking that can lead to protein degradation or epitope masking respectively); (3) many antibodies used for rodent studies are not suitable for human tissue, as the epitopes of proteins in human and mouse tissue can differ.

Another challenge is scalability. Numerous steps in the procedure, such as sample processing time, image acquisition time, data size, and processing time, scale linearly. Consequently, because human organs are much larger than rodent organs, there is a great need to develop high-throughput microscopes with very long working distances and more efficient ways to handle big data.

## What kinds of biomolecules can be labeled in cleared tissue?

Most TC techniques are compatible with protein IHC [11, 24]. Nonetheless, immunohistochemical labeling of proteins via antibodies is a lengthy process that generally takes a few days as the antibodies diffuse slowly through the thick tissue. In PACT, to help antibody penetration and to ensure high-quality and uniform staining across large cleared organs, the organ is often bisected or sliced into thick sections (~2 mm). Systemic delivery of antibodies and the use of small molecules such as nanobodies are additional options to circumvent antibody penetration issues without sectioning the tissue. Recently it was reported that the use of a small amount of detergent during the antibody incubation time could improve penetration and labeling uniformity [25].

**Fig. 2.**
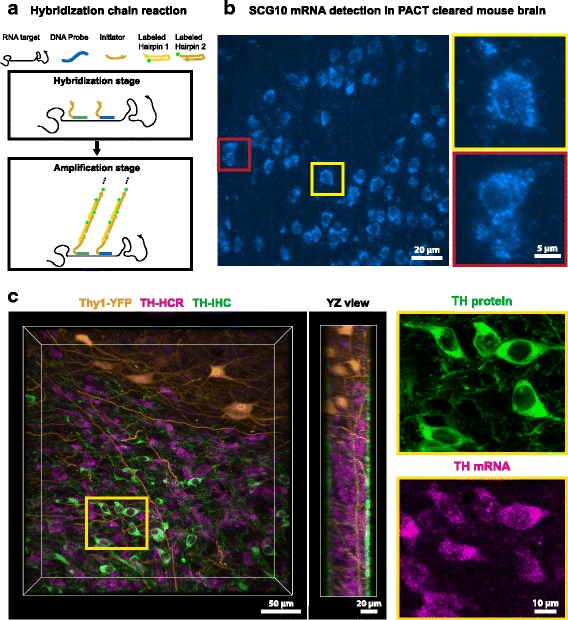
RNA detection at depth by hybridization chain reaction. **a** The probe hybridization step and the HCR amplification step. In the hybridization step, ~ 10–20 DNA probes bind to the target RNA. These probes carry the initiators for the HCR amplification step. In the HCR amplification step, each initiator triggers the binding of two hairpin types in order to amplify the fluorescence signal. **b** Detection of SCG10 mRNA transcripts in thick and cleared mouse brain 136 μm from the tissue surface. Adapted from [26]. **c** Simultaneous labeling of mRNA transcripts and proteins using HCR and antibody staining (IHC) in uncleared tissue expressing endogenous fluorescent proteins (YFP). During the same period of time (18 h), HCR achieved uniform labeling of mRNA transcripts through the tissue section, whereas protein IHC only reached a shallow depth (~ 10 μm) below the surface (YZ view). The tissue section was soaked in refractive index matching solution for imaging up to 50 μm deep into the tissue. Magnified inserts: note a subset of cells show TH mRNA but not TH protein signal

Single-molecule RNA FISH is another labeling option in cleared tissue, first demonstrated in Yang et al. [10]. The ability to label RNAs is crucial to providing a ‘snap-shot’ of cell state at a precise time point. Recent studies have shown that RNA retention can be improved in hydrogel-based clearing methods by adding chemical linkers or an additional post-fixation step [26, 27]. However, because it produces a weak signal relative to the background, FISH is only applicable to shallow depths within thick cleared tissue samples [10]. Hybridization chain reaction (HCR) is used to overcome this barrier and can boost the fluorescence signal by an order of magnitude [26–29]. HCR is a non-linear amplification method that uses DNA probes designed to bind the target RNA and trigger self-assembly of fluorophore-conjugated DNA hairpins (Fig. 2a). When HCR is combined with TC, it is possible to detect single mRNA transcripts in 0.5 mm-thick mouse brain tissue (Fig. 2b) [26] and rRNA in sputum samples [30]. As the labeled RNA photo-bleaches relatively fast, imaging thick cleared tissue benefits from using light sheet microscopy and/or anti-fade buffer [26]. Recently, sequential hybridization and barcoding techniques have enabled the transcriptional profiling of more than 100 genes from thousands of single cells, while maintaining their native environment in the tissue or cell culture [31, 32]. Although these results were not obtained in a cleared tissue, they provide the technical framework to achieve highly multiplexed RNA profiling of individual cells in thick cleared tissue. Furthermore, the smaller size of the probes facilitates diffusion into deep tissue, thus accelerating the tissue-staining stage in comparison to IHC (Fig. 2c). In addition, applying both FISH and IHC to the same tissue is also feasible when labeling limitations arise, such as limited antibody availability or RNA probe design difficulties due to sequence homology [33] (Fig. 2c).

## What are the preferred imaging devices for tissue clearing?

Standard confocal microscopy is the most convenient option for high-resolution and small-volume image acquisition of cleared tissue [34, 35]. However, it is impractical to image entire organs with a confocal microscope as the microscope scans the sample point by point, resulting in extremely long acquisition times and sample photobleaching. To overcome these obstacles, we recommend using light sheet microscopy (LSM). LSM is ~ 10–100 times faster than a typical confocal microscope because it images an entire plane at a time [36]. Also, since LSM illuminates only one plane at a time (the plane that is being acquired), photobleaching is minimized [36, 37]. Imaging using LSM is a big time-saver—entire organs can be imaged in only a few hours. Nevertheless, the expected resolution in traditional LSM is inferior to confocal microscopy [38]. Recently, customized light-sheet set-ups with improved performance have been introduced for TC. These microscopes utilize long working distance objective lenses (~8 mm), adaptively compensate for refractive index variations to reduce aberrations, and synchronize the illumination beam with the camera readout to improve the signal-to-noise ratio [17, 34].

## Can we detect changes in neuronal activity in cleared tissue?

It is possible to detect changes in neuronal activity via the use of TC methods. TC captures a snapshot of the tissue at a moment in time, which affords the inference of neuronal activity at that time. An increase in neuronal activity can be identified either by staining for immediate early genes (IEGs), such as c-fos [24], or by utilizing a genetic system where active neurons are permanently labeled [39]. In the latter, a behavioral experiment is conducted during a ~ 12-h period of tamoxifen induction that enables *Arc*, an IEG, to drive fluorescent protein expression [39]. This requires the use of a tamoxifen-dependent *Cre* recombinase (CreER) [40] that can remove floxed-stop sites to drive fluorescence reporter expression when tamoxifen is delivered systemically. These methods offer insight into brain-wide information processing. An additional option that might provide a faster readout is RNA FISH. Given that mRNA transcription occurs prior to protein translation, RNA FISH could detect upregulation of IEG mRNA immediately after a behavioral experiment. However, TC techniques cannot measure real-time neuronal activity.

## What tools are available to monitor cell state and physiological activity in live tissue?

Real-time monitoring of cellular physiology is an essential component of systems and behavioral neuroscience studies. Classic techniques use implanted electrodes to monitor electrical activity and can determine neuronal firing with high temporal precision. However, because electrophysiological signatures are similar among cell types yet can also widely vary within specific populations, it is difficult to determine the precise neuronal type from which one is recording. The development of genetically encoded calcium indicators and voltage indicators (GECIs and GEVIs, respectively) has been instrumental in overcoming this obstacle by relying on changes in fluorescent protein intensities to monitor activity-dependent calcium dynamics or changes in membrane voltage [41, 42]. Once GECIs or GEVIs are expressed in a desired neuronal population, fiber photometry or two-photon imaging is used to detect the activity of large neuronal ensembles in vivo. Although the use of microelectrode arrays has increased the number of units that can be recorded simultaneously [43], standard electrophysiological techniques still have a lower throughput than these genetic methods. While the temporal precision may not be on a par with those of electrode recordings, activity indicators are constantly being improved for faster and optimized performance [44]. The development of these optogenetic reporters of in vivo neuronal activity has complemented a separate class of optogenetic tools used to inhibit and excite neuronal populations.

## How have optogenetic tools evolved to study diseases?

Since a microbial light-gated ion channel was first adapted for use in mammalian cells, countless efforts have been made to modify the protein or discover novel channels to address different experimental needs. This has resulted in effectors with improved sensitivity, broadened spectral properties, and altered ion conductance to control neuronal activity, and their use has become a staple in systems neuroscience research. *Channelrhodopsin* (*ChR2*), a light-activated cation channel, is used ubiquitously in neuroscience research to control neuronal excitation with blue light [45]. *Halorhodopsin* (*NpHR*), meanwhile, is a light-gated chloride pump that is activated in response to yellow-green light and can inhibit neurons [46]. Both *ChR2* and *NpHR* have been used in numerous studies to dissect neurobiological circuits relevant to many disorders, including Parkinson’s disease [47], mood disorders [48], and drug addiction [49]. Many other optogenetic effectors have been characterized and groups are tirelessly working to modify and engineer novel molecules to address growing research needs [50]. This includes variants with ultrafast control and increased currents [51]. Describing the advantages of each variant is beyond the scope of this Q&A; however, several comprehensive resources are easily accessible [52, 53].

Delivery of both actuators and sensors to the brain is not without challenge as the widely used method of direct intracranial injection only provides limited and non-uniform expression. The recent development of novel viral capsids that can effectively traverse the blood–brain barrier has made it possible to non-invasively deliver constructs to the brain via systemic injection [54, 55]. Systemic gene delivery eliminates confounds that might arise from direct injections into the brain and was recently used successfully for real-time monitoring of large-scale cortical activity relevant for proper olfaction [56] and vision [57]. Coupled with opsin variants that are activated by far-red light [58], which can more readily penetrate tissue, it is possible in principle to manipulate neuronal activity without major surgery and the associated confounding factors.

## Can optogenetic techniques be used to study the peripheral nervous system?

Unlike in the brain, where neurons are densely clustered in well-defined nuclei, neurons in the peripheral nervous system (PNS) are contained in separated ganglia connected by a complex, far-reaching network. Separately, the spinal cord, a critical modulator of sensory and motor function, is delicate, hard to access through bone, and easily affected by movement. These factors complicate both opsin delivery via local injection and photoactivation via optical fiber placement in these regions. New approaches are being developed to improve opsin delivery to the spinal cord and PNS, including the use of novel viral capsids with high affinity for peripheral neurons, which can express opsins in the desired locations after systemic injection [54, 55]. Light delivery strategies must also be optimized for the spinal cord or peripheral tissue as they cannot easily be targeted with fiber optics. This has led to a broad range of tactics for peripheral illumination that include epidural fiber implants [59] and polymer-based optic cuffs [60]. Such developments can bridge the current gap between optogenetic CNS and PNS studies.

## What are the preferred imaging devices for optogenetics?

Besides the traditional fluorescence microscope, several imaging devices/systems are available for acquiring calcium indicator signals, such as fiber-photometry, two photon microscopy, implantable miniature microscopes, and macroscopes [61]. Fiber photometry operates by recording population activity through a relatively large (~ 400 μm diameter) ceramic optical fiber that is implanted above an area of interest in the brain. During the recording interval, subjects are freely behaving, and multiple color channels can be simultaneously recorded using the same fiber. While fiber photometry is an easy-to-use and relatively economical system, it lacks spatial resolution because the recorded signal is an accumulation of the fluorescence intensity from activity of the entire population. Two-photon microscopy mitigates the spatial resolution drawback of fiber-photometry and is capable of recording neuronal activity with sub-cellular resolution and an excellent signal-to-noise ratio. Two-photon microscopes can scan volumes of interest using a piezo-controlled objective. To extend the working distance of the objective lens and to image deep brain areas, two-photon microscopes can also image through implanted GRIN lenses. However, two-photon microscopy requires substantial capital investment and animals must be head fixed, thus restricting the scope of behavioral experiments that can be conducted. To address this limitation, light-weight (< 3 grams), miniature fluorescence microscopes have been developed and can be directly implanted on top of a mouse’s head [62, 63]. Following implantation, the miniature microscope records real-time neuronal activity from freely moving animals at single-cell resolution. Miniature microscopes are commercially available and can also be assembled with the guidance of on-line tutorials provided by the miniscope open-source community [64]. The technology behind miniature implantable microscopes is rapidly developing, and a two-photon version was recently introduced in freely behaving animals [65]. Finally, when studying superficial brain areas using a cranial window, an extremely wide field of view of up to 20 mm in diameter can be recorded using a macroscope, which can reveal cortex-wide activity patterns with a small compromise in resolution [56, 66].

## What is the typical size of a dataset, and how do you manage them?

Both TC and live imaging techniques generate a large number of images in order to create a 3-D volume or time-series dataset. Three-dimensional imaging of cleared tissue usually creates tens to hundreds of gigabytes (GB) of data for a single experiment, thus requiring not only large computational power for 3-D visualization, but also extensive storage capacity. Sacrificing resolution in order to reduce the acquired dataset dimensions to a manageable size is a reasonable trade-off, as long as the biological question can be addressed within the lower-resolution dataset. Similarly, time-series image datasets acquired from calcium-imaging experiments can be post-processed and converted to a compact format, which contains only the average intensity per cell in time. To manage and to access the acquired data easily, a workstation with enough memory to handle big datasets (hundreds of GB) and with secured network-attached storage (NAS) is a practical option.

Sharing large datasets with distant collaborators is another challenge. Commercial cloud services provide a convenient solution, even if the data transfer speed is relatively slow and the storage space is generally limited. Omero [67], a server system specifically designed for scientific images, could be another option for this purpose.

## What are the tools and challenges for data analysis?

One difficulty in analyzing TC data arises from optical limitations in 3-D imaging, such as chromatic aberrations in multi-colored images, which requires post-processing of the data. ImageJ (FIJI) [68], an open-source platform for image processing, includes a suite of useful plugins such as a 3-D viewer, versatile file-format converters, and image filters. Commercial data visualization software, such as Imaris or Amira, can render large 3-D volumes seamlessly, although they only provide simple functions for processing 3-D images. None of the above software can stitch a large volume that is composed of multiple tiles, and the open-source tool TeraStitcher [69] is a good solution for this purpose. For satisfying stitching results, at least 10% overlap between tiles is required.

**Fig. 3.**
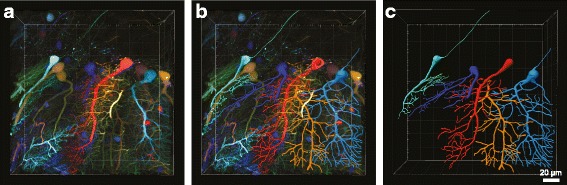
Viral-assisted spectral tracing of mouse cerebellum. **a** Multi-color neurons, labeled with a two-component viral delivery system. Two viral vectors carrying ssAAV-PHP.eB:TET-DIO-XFP (XFP = mNeonGreen, mTurquiose2, or mRuby) and ssAAV-PHP.eB:ihSyn-tTA-WPRE were injected into the retro-orbital sinus of an adult mouse. After 3 weeks of expression the animals were sacrificed and the brain was cut into 300 μm thick sections. The section was optically cleared in refractive index matching solution (RIMS) and then imaged using a confocal microscope. Refer to [55] for details. **b** Using the 3-D volume data, five Purkinje cells were manually traced in NeuTube and their traces overlaid on the 3-D volume. **c** Traces of the five Purkinje cells from **b**

Cell morphology is of particular interest in neuroscience owing to its association with function and neural diseases. However, 3-D reconstruction and digitization of fine neuronal morphology remains challenging. One reason is that cells in the nervous system are densely packed, making it difficult to distinguish one from another. Despite many attempts to solve this problem, automated tracing of neuronal morphology is highly limited to specific cell types and labeling methods. For manual tracing, the Imaris module, FilamentTracer, is useful. Among open-source tools, neuTube [70] is another good option, though it only supports grayscale images (Fig. 3).

Although there are various software tools that attempt to tackle these numerous challenges, each of them typically focuses on one specific task—such as stitching, visualization, or deconvolution—and receives a distinct file format as input, requiring file-type conversions for further processing. Consequently, sequential use of these tools is a tedious and bewildering process. There is a need for a unified computational framework or a shared file standard so that these software tools can interface in a seamless manner.

## What are the examples beyond neuroscientific applications?

Beyond the brain, TC techniques have been applied to other organs [11] such as the skin [10], heart [71], pancreas [10, 72], and tongue [73] to reconstruct their 3-D structure and identify anatomical changes under abnormal conditions. For example, TC was used to study cancer metastasis at cellular resolution in whole-body cleared mice [15, 74], as well as to characterize axon regeneration and glial response after injury in the spinal cord [16].

In addition, bone, which has a unique composition of both hard (mineral) and soft (bone marrow) tissue [75], can also be rendered transparent with a tailored clearing protocol, Bone CLARITY [17]. Bone CLARITY uses EDTA to remove minerals that make the bone rigid and 8% SDS to remove lipids, the primary source of light scattering in biological tissue. This technique is also able to maintain the native fluorescence of reporter mouse lines. When paired with light sheet microscopy, Bone CLARITY can detect rare and non-uniformly distributed stem cells within the bone marrow at single cell resolution in the long bones and vertebral bodies of adult mice. Furthermore, this provides a way to assess therapeutic candidates for osteoporosis.

The ability of hydrogel-based TC techniques to lock molecules in place also enables us to investigate the spatial distribution of pathogens in specimens. Our recent study applied the PACT technique to capture sputum collected from cystic fibrosis patients and showed in situ characterization of bacterial physiology [30]. Spreading patterns of viruses, such as HIV-1, have also been studied by applying TC techniques to lymphoid tissue [76]. These examples suggest the potential applications of TC techniques to prokaryotic organisms and the diagnosis of infectious diseases.

Although optogenetic tools have been most widely used to further our understanding of neuronal function, they have also been used to manipulate the activity of other cell types such as astrocytes [77] and cardiomyocytes [78]. Additionally, several effectors have been engineered to manipulate other biological events, including intracellular transport [79], signaling pathways [80], gene expression [81], and protein assembly [82].

## What are the future directions of tissue clearing and optogenetics?

Applicable from the soft brain to the hard bone, TC methods allow us to study the structure and connectivity of whole organs in unprecedented resolution and detail. As mentioned above, the handling, management, and analysis of TC datasets remains an unsolved challenge, hindering biological discoveries. The quality, uniformity, and cost of antibody labeling are additional challenges for TC. Thus, addressing the need for a unified computational framework and low-cost small-molecule labeling methods is paramount for the successful integration of TC techniques into the life sciences and medicine.

The combination of discrete expression and high temporal resolution has led to the use of optogenetics to study the brain; however, there is much to be addressed in the future of the field. In particular, its current implementation is invasive as it often requires the injection of viral vectors for cell-specific expression, implantation of fiber optics to target a specific region, and chronic optical stimulation. Tools are rapidly being engineered to solve these issues. Recently developed novel capsids allow viral particles to traverse the blood–brain barrier and achieve high gene expression throughout the brain, thus beginning to overcome the barriers that stand in the way of efficient, non-invasive gene delivery [54, 55]. Opsins are also continuously being engineered to address obstacles concerning light delivery. Recent variations of the *Channelrhodopsin* protein include a variant that is activated at far-red shifted wavelengths, which enables activation at greater tissue depths, and a bistable variant that only requires brief pulses of light to switch between ‘on’ and ‘off’ states [83]. Novel environments are also being developed to take advantage of new opsins, such as those that allow complete, wireless photostimulation [84].

Emerging from the field of neuroscience, TC and optogenetics now constitute a powerful approach to the study of general biological questions and diseases by enabling the control and measurement of functional changes in living tissue [85], followed by cellular and molecular phenotyping at the whole-organ level.
